# Vegetables and legumes in new Australasian food launches: how are they being used and are they a healthy choice?

**DOI:** 10.1186/s12937-018-0414-2

**Published:** 2018-11-09

**Authors:** Beth Gilham, Ramon Hall, Julie L. Woods

**Affiliations:** 1 Institute for Physical Activity and Nutrition (IPAN), School of Exercise and Nutrition Sciences, Deakin University, Geelong, 3220 Australia; 2 Centre for Advanced Sensory Science, School of Exercise and Nutrition Sciences, Deakin University, 221 Burwood Highway, Burwood, VIC 3125 Australia

**Keywords:** Food supply, Vegetables, Legumes, Pulses, Packaged foods

## Abstract

**Background:**

Despite the health benefits of regular vegetable and legume consumption, on average Australians are consuming only half of the recommended daily intake. The reasons for this low consumption are complex, and are particularly driven by societal shifts towards convenient and ready-to-eat meal options. It is currently unknown how legumes and vegetables are being utilised in food products within the Australian context, and the nutritional value or level of processing of these products.

**Methods:**

The Mintel Global New Food Database was used to identify all new products launched between May 2012 and May 2017 in Australasia which at least 0.5 serves of vegetables and/or legumes per recommended serving. Eligible products were coded using the NOVA food classification system and the Healthy Choices guidelines, and were categorized by the researchers based on the type and proportion of vegetable and legume ingredients used.

**Results:**

Overall, 1313 products were identified, which contained a median of 55% vegetable and legume ingredients (IQR = 45%). This translated to approximately 1 (IQR = 1) serves of vegetables and legumes per recommended serving of the products. The product launches were most likely to be classified as an ‘amber’ choice, and be classified as either ‘processed’ or ‘ultra-processed’. Vegetables and legumes were mainly found in the form of new prepared meals, soups or whole vegetables products, however there were some more innovative uses of these ingredients, such as yoghurts and pastas.

**Conclusions:**

Most of the new products currently released onto the Australian market which contain vegetable and legume ingredients do not provide meaningful amounts of these ingredients, and tend to be highly processed and unhealthier options. A multi-faceted approach is needed to improve vegetable and legume consumption, which includes improving the availability of products which help consumers to meet vegetable and legume consumption recommendations. Future research should consider the acceptability of these products to consumers, and the barriers for food manufacturers in creating products with a higher amount of vegetables and legumes.

## Introduction

In a society where poor diet habits are one of the largest contributor to the burden of disease [1], it is vital that we find ways to improve population-level consumption of healthier diets. Vegetables and legumes, such as chickpeas, lentils, lupins and beans, form a key part of a healthy diet, and regular consumption of these foods has been shown to have a positive impact on improving risk factors for a range of non-communicable diseases [2–4]. Despite being the most prominent food group in the Australian Dietary Guidelines, vegetables and legumes are one of the most under-consumed food groups in the Australian diet [5]. On average, most Australians are currently consuming only half of the recommended five serves (1 serve = 75 g) from the vegetable and legumes group per day [5]. This poor consumption has been linked to a range of barriers in the Australian context, such as preference for other foods or dislike of vegetables [6, 7], the consumer perspective that they consume more vegetables than they actually do [6] and poor access to, and higher cost of fresh produce, particularly in rural or outer-metropolitan areas [7–10]. Legumes in particular are not well recognised or understood by consumers [11, 12], which has been described as a significant barrier to consumption and led to the creation of the ‘International Year of Pulses’ campaign in 2016 by the Food and Agricultural Organisation of the United Nations [13].

Societal changes and the globalisation of the food supply have led to a shift in the types of food people eat, as well as the way they are eaten, with consumers increasingly choosing convenient food options that require minimal preparation such as ready-to-eat meals and snack foods [14, 15]. Concurrently, we are consuming more discretionary foods (foods which do not form a key part of the diet, such as chips, chocolate and muesli bars) and products which have been ultra-processed [5, 16]. These products tend to be energy dense and nutrient poor, but are typically considered more convenient and palatable compared to fresh, minimally processed, healthier options [17, 18]. The modern food environment offers many convenient options, however it is unknown whether these products contain healthier ingredients such as vegetables and legumes, and the relative nutritional value of these products.

A recent study by Spiteri et al. found that only 3% of all new products launched onto the Australian market in 2015 were categorized as ‘fruit and vegetables’. Of these ‘fruit and vegetables’ products, were classified as ‘green’ (ie. the healthiest choice) using the Healthy Choices guidelines [19]. As expected, there was considerable overlap between the products in this study [19] and the current study; however the study by Spiteri et al. was conducted for one year only, and only counted fruit and vegetables categorized in the ‘fruit and vegetables’ category, not in mixed meals or other product categories. To date, there has been a dearth of information relating to the measurement of the use of vegetables and legumes in new food products in Australia. Likewise, there is no indication of whether these new products, despite containing healthier vegetable and legume ingredients, are a healthy and/or minimally processed option. This information is essential to establish a baseline understanding of the situation and products currently available. It is particularly important for the food industry in order to establish the current use of vegetable and legume ingredients in food products, and to identify new innovations in this area.

The objective of this study was to examine the use of vegetables and legumes in new food and beverage products released in Australia and New Zealand over a 5-year time period based on i) proportion of vegetable and legume ingredients; ii) number of serves of vegetables and legumes; iii) type of food or beverage product; iv) nutritional quality and; v) level of processing.

## Materials and methods

### Data collection

This study identified new food and beverage launches in Australia between May 2012 and May 2017 using the Mintel Global New Product Database (Mintel GNPD). The Mintel GNPD is a large industry database which catalogues all new packaged food and beverage launches in 60 economies worldwide, including Australia and New Zealand [20]. Each entry provides detailed product information, such as price, ingredients, claims made and nutritional information, as well as photographs of all sides of the packaging. Approximately 33,000 new product launches are added to the entire database per month, and the products available are predicted to represent 75–80% of all new launches in countries where data is collected [20, 21].

New food and beverage products launched between May 2012 and May 2017 in Australia, which contained at least 0.5 serves of vegetables and legumes per recommended serving were included in this study (Table 1). Vegetables are the edible portion of a plant, either in the form of a raw product or processed in a way that retains the bulk of the raw product [22]. In general, vegetables are an excellent source of fibre and a wide range of essential vitamins and minerals. There is a wide variety of different vegetables available in Australia, with the main examples being green leafy vegetables (such as spinach), Brassica vegetables (such as broccoli), gourd vegetables (such as pumpkin), edible plant stems (such as asparagus) and Allium vegetables (such as onion or garlic) [23]. This study also included vegetables such as tomatoes and potatoes, which are not universally classified as vegetables, but are included in the Australian Dietary Guidelines [23]. Vegetable and legume flours and juices were included in addition to raw or whole versions.

**Table 1:** Inclusion and exclusion criteria

Included	Excluded
▪ New food and beverage products (May 2012 – May 2017) ▪ < 0.5 approximate serves of vegetables and legumes ▪ Tomatoes, potatoes ▪ Vegetable and legume juices and flours	▪ Soybeans and peanuts ▪ Vegetable oils ▪ Corn flour

Products were excluded if the sole vegetable or legume ingredient did not resemble the nutritional properties of the whole ingredient, such as vegetable oils. While corn was included as a vegetable, corn flour was considered as a grain due to its low fibre and micronutrient content [24], and therefore products containing corn flour as the sole vegetable or legume ingredient were excluded. While both soybeans and peanuts are part of the legume family, they are usually considered as oilseeds due to their high lipid content [25], and therefore products where these were the only vegetable or legume ingredient were not included in the analysis. Once calculated, products with less than 0.5 approximate serves of vegetables and legumes were dropped.

The Mintel GNPD search was conducted on 23rd June 2017, using the search parameters listed in Table 2. Additional searches were conducted using the same parameters and specific key words in order to identify products which may not be consistently classified in the vegetable category (‘potato’, ‘pea’, ‘tomato’, ‘chickpea’, ‘kidney’, ‘lentil’). The results of all searches were exported to Microsoft Excel, where duplicate results and excluded products were dropped.

**Table 2:** Search strategy for Mintel Global New Product Database

Search variables	Parameters
Country	Australia
Date published	Between May 2012 and May 2017
Ingredient search	“Vegetables and Vegetable Products and all child ingredients”
Mintel GNPD categories included	‘Baby food’, ‘bakery’, ‘breakfast cereals’, ‘dairy’, ‘desserts & ice cream’, ‘fruit & vegetables’, ‘meal & meal centers’, ‘processed fish/meat/egg products’, ‘savoury spreads’, ‘side dishes’, ‘snacks’, ‘soup’, ‘sweet spreads’, ‘juice drinks’, ‘other beverages’, ‘RTDs’, ‘sports & energy drinks’, ‘sauces & seasonings’ (sub-categories: ‘cooking sauces’, ‘other sauces & seasonings’, ‘pasta sauces’, ‘pickled condiments’, ‘table sauces’)
Mintel GNPD categories excluded	‘Chocolate confectionary‘, ‘sugar & gum confectionary’, ‘sweeteners & sugar’, ‘alcoholic beverages’, ‘carbonated soft drinks’, ‘hot beverages’, ‘water’, ‘pet food’, ‘sauces & seasonings’ (sub-categories: ‘dressings & vinegar’, ‘mayonnaise’, ‘oils’, ‘seasonings’, ‘stocks’)

Foods in Mintel GNPD are classified into 22 main categories, with additional sub-categories: baby food, bakery, breakfast cereal, carbonated soft drinks, chocolate confectionery, dairy, desserts and ice cream, fruits and vegetables, hot beverages, juice drinks, meals and meal centres, other beverages, processed fish, meat and egg products, sauces and seasonings, savoury spreads, side dishes, snacks, soup, sports & energy drinks, sugar & gum confectionery, sweet spreads and water. Some of these categories were excluded from the search as they were inappropriate for this study. This included categories which are considered condiments (such as vinegar, dressings and sugar/sweetener), discretionary foods with small serving sizes (such as chocolate confectionary) and drinks which are not suitable for general consumption (such as alcoholic beverages). Sensitivity analyses including these categories revealed no reportable change to the results.

### Categorization based on type and proportion of vegetable and legume ingredients

All products were initially classified as containing ‘vegetables-only’, ‘legumes-only’ or ‘both legumes and vegetables’. All other variable categories (food category, food sub-category) were directly sourced from Mintel GNPD as described above.

The approximate proportion of vegetable and legume ingredients was determined based on the proportion listed in the ingredients list. Where no percentage was listed for one or all of the legume and vegetable ingredients in the ingredients list, the proportion was estimated based on similar products, and the proportion of other ingredients in the product. This percentage was converted to grams of vegetable and legume ingredients, based on the recommended serving size of the product. The equivalent number of serves from the vegetables and legumes group was then determined, where one serve was equal to 75 g of vegetables and legumes as per the Australian Dietary Guidelines [23]. The number of vegetable and legume serves was rounded down to the closest 0.5 serve to account for potential loss of product through preparation or wastage, and to prevent overestimation of the vegetable and legume content.

### Categorization based on nutrition quality and level of processing

The nutritional quality of products was determined using the Victorian Government “Healthy Choices” framework, which classifies products as ‘green’ (healthiest choice), ‘amber’ (choose carefully) or ‘red’ (limit) based on several attributes, such as nutritional profile and serving size [26]. Level of processing was determined using the NOVA food classification system, which classifies products as ‘unprocessed or minimally processed foods’, ‘processed culinary ingredients’, ‘processed foods’ or ‘ultra-processed food and drink products’ [27].

Initial coding of the products using both classification systems was conducted by one researcher (BG). A random sample of 5% of the total products was cross-checked by the two other researchers (JW, RH), as per similar literature in this area [28]. Where discrepancies were identified, these were discussed as a group to determine the final classification.

### Statistical analysis

All analyses were conducted using SPSS 23 (SPSS Inc., Chicago, SPSS for Windows, version 23). Frequency tests were conducted to determine the number of products within each category, and median values with interquartile ranges were calculated to determine the average proportion of vegetable and legume ingredients, and number of vegetable and legumes serves in each product.

### Ethics

This study was exempt from ethics approval as the analysis was conducted on food products and did not directly use human or animal data.

## Results

Between May 2012 and May 2017, 1313 products were released in Australia which contained greater than 0.5 serves of vegetables or legumes per standard serve. This constituted 6% of the total food and beverage launches in the period (*n* = 21,111). The majority of these products contained vegetables only (*n* = 1013; 77%), while a smaller proportion of products contained legumes only (*n* = 108; 8%) or both vegetables and legumes (*n* = 192; 15%).

### Proportion of vegetable and legume ingredients

The proportion of vegetable and legume ingredients in each product varied between 11 and 100%, with a median of 55% (IQR = 45%). Fifty-eight percent of products contained over 50% vegetable and legume ingredients (*n* = 764), and 24% contained greater than 80% vegetable and legume ingredients (*n* = 319).

### Number of vegetable and legume serves

**Fig. 1:**
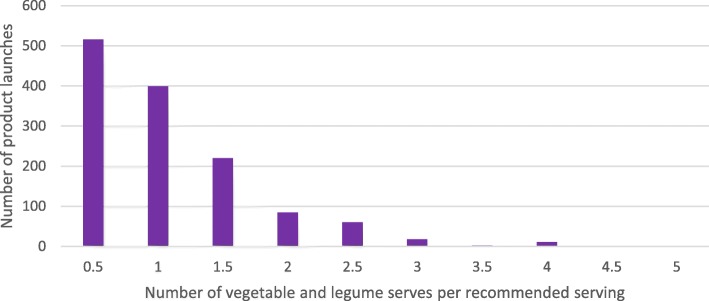
Number of new product launches by number of vegetable and legume serves

New product launches containing vegetable and legume ingredients had a median of 1 (IQR = 1) serve of vegetables and legumes per recommended serving size of the product (Fig. 1). Forty-nine percent (*n* = 915) of products provided between 0.5 and 1 serve of vegetables and legumes per recommended serve of the product, while only 14% (*n* = 178) contained 2 or more serves per recommended serve. One product, a tomato and capsicum soup, provided approximately 5 serves of vegetables per 430-g serve.

### Type of food or beverage product

New product launches containing vegetable and legume ingredients were most likely to be from the ‘meals and meal centers’ category (*n* = 362; 28%), which included ready-to-eat prepacked meals such as curries or lasagnes (Table 3). Other common launch categories containing vegetable and legume ingredients included ‘fruit and vegetables’ (*n* = 259; 20%), ‘soup’ (*n* = 238; 18%) and ‘side dishes’ (*n* = 124; 9%). ‘Sauces and seasonings’ in large serving sizes, such as pasta sauces, cooking sauces or pickled condiments, made up 11% of the launches (*n* = 138).

**Table 3:** Number and proportion of product launches in each food category and sub-category

Food category	Food sub-category	# products	% total products	Mean # serves
Baby Food	Baby Fruit Products, Desserts & Yogurts	1	0%	1.5
	Baby Juices & Drinks	1	0%	0.5
	Baby Savoury Meals & Dishes	56	4%	0.9
	Other Baby Food	1	0%	0.5
	Total	59	4%	0.9
Bakery	Baking Ingredients & Mixes	3	0%	0.5
	Bread & Bread Products	1	0%	1.0
	Total	4	0%	0.6
Dairy	Spoonable Yogurt	1	0%	0.5
	Total	1	0%	0.5
Fruit & Vegetables	Fruit	1	0%	1.0
	Vegetables	258	20%	1.1
	Total	259	20%	1.1
Juice Drinks	Juice	53	4%	1.4
	Nectars	14	1%	1.3
	Total	67	5%	1.4
Meals & Meal Centers	Instant Noodles	3	0%	0.8
	Instant Pasta	5	0%	1.0
	Meal Kits	40	3%	1.0
	Pastry Dishes	15	1%	0.5
	Pizzas	5	0%	1.0
	Prepared Meals	265	20%	1.1
	Salads	22	2%	1.0
	Sandwiches/Wraps	7	1%	0.5
	Total	362	28%	1.0
Other Beverages	Meal Replacements & Other Drinks	4	0%	0.6
	Total	4	0%	0.6
Processed Fish, Meat & Egg Products	Fish Products	5	0%	0.5
	Meat Products	3	0%	0.5
	Meat Substitutes	14	1%	0.8
	Poultry Products	2	0%	0.8
	Total	24	2%	0.7
Sauces & Seasonings	Cooking Sauces	36	3%	0.7
	Pasta Sauces	95	7%	1.1
	Pickled Condiments	7	1%	0.6
	Total	138	11%	0.9
Savoury Spreads	Dips	7	1%	0.5
	Savoury Vegetable Pastes/Spreads	1	0%	0.5
	Total	8	1%	0.5
Side Dishes	Pasta	20	2%	0.8
	Potato Products	70	5%	1.1
	Rice	4	0%	0.5
	Stuffing, Polenta & Other Side Dishes	30	2%	0.7
	Total	124	9%	0.9
Snacks	Bean-Based Snacks	1	0%	0.5
	Corn-Based Snacks	1	0%	0.5
	Hors d’oeuvres/Canapes	11	1%	0.7
	Potato Snacks	8	1%	0.5
	Vegetable Snacks	4	0%	0.5
	Total	25	2%	0.6
Soup	Dry Soup	40	3%	1.0
	Wet Soup	198	15%	1.5
	Total	238	18%	1.5
	Total	1313		1.1

Table 3 shows that the highest proportion of products was in the sub-categories ‘prepared meals’ (*n* = 265; 20%) and ‘vegetables’ (*n* = 258; 20%). In some food sub-categories, there were only several new launches during the study period, however they were examples of more innovative uses of vegetable and legume ingredients. These included ‘spoonable yoghurts’ (yoghurt flavoured with sweet potato or pumpkin), ‘water-based ice lollies’ (frozen apple, carrot and beetroot juice), ‘stuffing, polenta and other side dishes’ (roasted, ready-to-eat, blended vegetable and legume ‘delights’), ‘pasta’ (pastas made with legume flour) and ‘meal replacements and other drinks’ (smoothie bases containing broccoli, beetroot and spinach). There were several examples of more convenient products containing vegetables and legumes, including zucchini ‘pasta’, pre-cut vegetables, and microwavable rice products.

Additionally, there were some innovations in the ‘bean-based snacks’ sub-category, in the form of chips made from beans and rice, snacks made from roasted chickpeas, and muesli bars with popped chickpeas. Similarly, creative innovations in the ‘vegetable snacks’ sub category included crackers made from crushed zucchini. These snacks were not included in the final analysis due to their small serving size, however are worth noting for some potential future uses of vegetables and legumes, if there is some consideration to nutritional value and level of processing.

### Nutritional quality and level of processing

Table 4 shows that the highest proportion of products was classified as ‘amber’ (46%). This included products such as soups, salads, vegetable juices, canned fish with tomato, frozen roasted vegetables (if baked rather than fried) and some ready-to-eat meals such as pastas and risottos. This was followed by ‘green’ options (the healthiest choices), which made up 28% of the products and included products such as canned baked beans, salads, fresh or frozen mixed vegetables, and several ready-to-eat meals composed primarily of vegetables and lean meat Approximately a quarter of product launches were classified as ‘red’ (*n* = 339), such as potato crisps, pizzas, some ready-to-eat meals, and soups made with cream and large quantities of salt. The majority of product launches were classified as either ‘processed’ (45%) or ‘ultra-processed’ (40%), with only 15% as ‘unprocessed or minimally processed’. No products were classified as ‘processed culinary ingredients’.

**Table 4:** Number and proportion of launches in each Healthy Choices or NOVA category

	Number of launches	Proportion of launches
Healthy Choices classification		
‘Green’	373	28.4%
‘Amber’	601	45.8%
‘Red’	339	25.8%
TOTAL	1313	
NOVA food classification		
‘Unprocessed or minimally processed’	196	14.9%
‘Processed culinary ingredients’	0	0%
‘Processed foods’	590	44.9%
‘Ultra-processed food and drink products’	527	40.1%
TOTAL	1313	

## Discussion

Products containing at least 0.5 serves of vegetable and legume ingredients per recommended serving represented 6% of new product launches between May 2012 and May 2017. On average, these products contained approximately 55% vegetable or legume ingredients, however this translated to only 1 serve of vegetable and legumes per recommended serving size. The product launches were most likely to be ‘meal and meal centers’, or ready-to-eat meals, were an ‘amber’ choice, and were classified as either ‘processed’ or ‘ultra-processed’.

There are many barriers to consumption of vegetables and legumes, which makes it a complex issue to tackle. Public health campaigns conducted both in Australia and internationally, such as the Western Australia “Go for 2 & 5” campaign, have aimed to increase awareness of the benefits of regular fruit and vegetable consumption and drive this change [29]. While these campaigns have been demonstrated to increase awareness and lead to modest increases in consumption in the short term, the majority have been unable to sustain increased vegetable consumption in the long term [29]. Given the complexity of the issue, a multi-faceted approach is most likely to be successful at driving increased vegetable and legume intake. In addition to improved education and public health campaigns to support the consumption of fresh produce, a food environment with a wide range of products containing vegetables and legumes could help to increase opportunities to meet the 5 serves per day recommendation. While fresh produce should be the main source of vegetables and legumes, processed or pre-packaged options can compliment this action by providing an additional opportunity for consumers to consume meaningful amounts of these healthier ingredients. The current evidence from this study indicates that with 1 serves of vegetables and legumes per serve, the new products currently being introduced onto the market are not yet supporting this.

Only 208 new products launched annually in Australia between 2012 and 2017 had legumes as a main ingredient. Less than 5% of the Australian population report consuming legumes or legume-based products, and the per capita consumption of legumes in Australia (2.9 kg per year) is well below the world average of 5 kg per year [30]. Legumes are unfamiliar to Australian consumers [11, 12, 30, 31], although some legumes, such as chickpeas, are becoming more common in products, such as hummus dip [30]. Food manufacturers in this market have the opportunity to improve consumer knowledge of legumes and how to use them, although this may be difficult if food manufacturers are also unfamiliar with legumes. The International ‘Year of Pulses’ campaign in 2016 aimed to address this lack of familiarity in all parts of the food supply, including consumers, manufacturers and growers [32]. The effects of this campaign have not yet been fully evaluated, and given that new product development and reformulation can be a lengthy process, it may be several years before more legume-based products appear on the market.

While this study indicates that vegetables and legumes are most commonly used in traditional ways, such as in pre-prepared meals (‘meals and meal centers’), soups and sauces, they are beginning to be used in a wider range of foods. These include pastas made from legume flour, yoghurts with pureed sweet potato and zucchini ‘pasta’. The incorporation of vegetables and legumes into commonly-eaten foods presents a new opportunity to introduce these ingredients to consumers, and could be included as complementary source of these ingredients to fresh vegetables and legumes. While a range of factors need to be taken into consideration, such as manufacturing methods, loss of key nutrients and chemical properties, healthier products such as pasta with up to one serve of vegetables and legumes per serve are possible [33, 34]. Whether consumers like these products, and consequently purchase and consume the product remains unknown, however it is promising that these types of products are beginning to become more widely available. Future research in this area could explore the acceptability and consumption of these new products, as well as the viability from a food manufacturing perspective.

The nutritional value of products with healthier ingredients, such as vegetables and legumes, is important. While vegetables and legumes provide a range of health and nutritional benefits in a less processed form, these benefits could be counteracted by adding excessive amounts of oil, salt and sugar during packaging or processing. This study found that the highest proportion of new product launches containing vegetable and legume ingredients were classified as ‘amber’ options. While these products are not considered the healthiest option, they typically contain less sodium, energy and fat compared to ‘red’ options, and are recommended to be consumed only occasionally as part of a healthy diet. Conversely, only 28% of launches were for products classified as ‘green’ options, or the healthiest options, which should make up the majority of the diet. This nutrient-base profiling system is not completely indicative of the value of foods in the diet: for example, a ‘red’ soup with a large proportion of vegetables may be a better choice compared to an ‘amber’ soup with less sodium but also less vegetables. However, when considered in conjunction with the other product classification systems used in this study, it can help to identify the general healthiness of new product launches, particularly in relation to products high in sodium and saturated fat, or those with large serving size. These findings indicate that overall, more work needs to be done on developing more healthier ‘green’ choices containing vegetables and legumes, while continuing to reduce the less healthy ‘red’ and ‘amber’ options, as well as promoting fresh vegetables and legumes.

The findings from this study show that the food market in Australia appears to be geared toward ‘ultra-processed’ foods, which may pose an issue to public health. ‘Ultra-processed’ products are industrial formulations with a large number of ingredients and additives designed to enhance hyper-palatability and mimic natural ingredients [27]. Previous literature has identified that ‘ultra-processed’ foods tend to be energy dense, nutritionally unbalanced and contain fewer beneficial nutrients such as protein and fibre [35, 36]. A focus on increasing the supply of minimally processed foods with legumes and vegetables, such as vegetable juice without additives, raw vegetables which have been peeled and chopped or vacuum-packed vegetables or legumes, is most likely to ensure the products are nutritionally beneficial and are more aligned with the Australian Dietary Guidelines. However, this may not always be possible from a manufacturing perspective. ‘Processed foods’ may provide a middle-ground, as these products are typically composed of a few core, minimally processed ingredients, with additives used to preserve the product or resist contamination only [27]. Where the use of oil, sugar and salt is minimized, healthier ‘processed’ products with a higher content of vegetables and legumes may help to address the previously discussed barriers to consumption, such as accessibility to fresh produce, and make these healthier ingredients more accessible to consumers.

### Limitations

Some limitations to this study should be noted. The proportion of vegetable and legume ingredients was based on the ingredients list provided, however it is possible that these were not completely accurate, as it is the prerogative of each manufacturer to supply this information. Although, with truth in labelling laws [37], manufacturers must ensure that the ingredient list is accurate and up-to-date. The use of proportion of vegetable and legume ingredients was also limited as it did not always give a true reflection of how the product contributes to overall vegetable and legume consumption. Despite 58% of the products containing greater than 50% vegetable and legume ingredients, when consumed as recommended, the majority of the products contained 1 serve of vegetables and legumes. The estimated number of vegetable and legume serves used the recommended serving size listed on the product, however it is unknown whether consumers adhere to this recommended size and hence consume the calculated vegetable and legume serves. Some product may be lost to wastage, leading to less serves, or conversely, consumers may actually consume more than one recommended serve in an eating occasion. Overall, this study provides a good indication of the new products available on the market, but unless they are purchased by consumers and remain on the market long-term, these products are unlikely to have a significant impact on vegetable and legume intake. Future research could explore the real-world consumption of these products, and identify the key enablers and barriers to the long-term success of these products in the marketplace.

## Conclusions

As a core part of a healthy diet, vegetables and legumes need to take more prominence in Australians’ diets, as well as the food products they have access to. A multi-faceted approach is needed to improve vegetable and legume consumption, however food manufacturers have a role to play by continuing to innovate and providing a larger variety of healthier options which provide more vegetables and legumes per recommended serving.

Most of the new products currently released onto the Australian market which contain vegetable and legume ingredients do not provide meaningful amounts of these ingredients, and tend to be highly processed and unhealthier options. While consumption of minimally processed foods is preferable from a nutritional perspective, this may not be possible for all people, and therefore there is a place for healthier, processed options containing whole legumes and vegetables to address a range of consumer needs and potentially increase consumption of this poorly consumed food group. This study provides a baseline understanding of the use of vegetables and legumes in the Australian context, which can be used to identify and drive new areas for healthier product development.
